# 

**Published:** 1880-08

**Authors:** 


VOL. 1. AUGUST, 1880 No. 8.

THE INDEPENDENT

### Practitioner:

A MONTHLY JOURNAL,

S.G.O. INDEXED

DEVOTED TO

***MEDICAL, SURGICAL, OBSTETRICAL, DENTAL and HYGIENICAL SCIENCE.***

EDITED BY
HARVEY L. BYRD, A. M., M. D.,
AND
BASIL M. WILKERSON, D. D. S., M. D.

*"Altera poscit opem res, et conjurat amice."*

BALTIMORE:
THE PRACTITIONER PUBLISHING CO.,
B. M. WILKERSON, PROPRIETOR,
No. 68 North Charles Street.

$2.00 Per Annum in Advance. - - Single Copies, 25 Cents.

ENTERED AT THE POSTOFFICE AT BALTIMORE, MD., AS SECOND-CLASS MATTER.

